# Combinatorial Effects of Terpene, Chenodeoxycholic Acid, and Ursodeoxycholic Acid on Common Bile Duct Stone Recurrence and Gallbladder Stone Dissolution

**DOI:** 10.3390/jcm13237414

**Published:** 2024-12-05

**Authors:** Min Je Sung, Sung Yong Han, Jong Hyun Lee, Tae In Kim, Dong Uk Kim, Chang-Il Kwon, Jae Hee Cho, Jung Wan Choe, Jong Jin Hyun, Jae Kook Yang, Tae Hoon Lee, Jungnam Lee, Sung Ill Jang, Seok Jeong

**Affiliations:** 1 Digestive Disease Center, CHA Bundang Medical Center, CHA University School of Medicine, Seongnam 13496, Republic of Korea; mj1744@cha.ac.kr (M.J.S.); endoscopy@cha.ac.kr (C.-I.K.); 2 Department of Internal Medicine, Pusan National University School of Medicine, Biomedical Research Institute, Pusan National University Hospital, Busan 49241, Republic of Korea; mirsaint@hanmail.net (S.Y.H.); keiasikr@nate.com (J.H.L.); zeitgeister88@daum.net (T.I.K.); amlm3@hanmail.net (D.U.K.); 3 Department of Internal Medicine, Gangnam Severance Hospital, Yonsei University College of Medicine, Seoul 06273, Republic of Korea; jhcho9328@yuhs.ac; 4 Department of Internal Medicine, Korea University Ansan Hospital, Ansan 15355, Republic of Korea; jwchoe@korea.ac.kr (J.W.C.); sean4h@korea.ac.kr (J.J.H.); 5 Department of Internal Medicine, Soonchunhyang University Hospital Cheonan, Cheonan 31151, Republic of Korea; c96396@schmc.ac.kr (J.K.Y.); taewoolee9@gmail.com (T.H.L.); 6 Department of Internal Medicine, Inha University Hospital, Inha University College of Medicine, Incheon 22332, Republic of Korea; jungnamlee@inha.ac.kr

**Keywords:** choledocholithiasis, recurrence, cholelithiasis

## Abstract

**Background:** Ursodeoxycholic acid (UDCA), chenodeoxycholic acid (CDCA) plus UDCA (C&U), and terpene are widely administered to prevent common bile duct (CBD) stone recurrence and dissolve gallbladder (GB) stones. We evaluated and compared the combined effects of these agents on CBD stone recurrence and GB stone resolution. **Methods:** This study included patients who underwent endoscopic retrograde cholangiopancreatography (ERCP) at six referral centers, retrospectively. A total of 940 patients who underwent cholecystectomy before or after CBD stone removal by ERCP were evaluated to assess CBD stone recurrence (the CBD recurrence cohort), and 98 patients with GB stones were assessed by abdominal or endoscopic ultrasonography before and 6 months after ERCP to evaluate GB stone resolution (GB cohort). Patients were divided into no-medication, single-agent treatment (UDCA, C&U, or terpene), or dual-agent treatment (terpene plus UDCA or C&U) groups for the analysis. **Results:** In the CBD recurrence cohort, baseline characteristics were similar in the three groups. CBD stone recurrence rates were 41.5%, 12.7%, and 9.8% in the no-medication, single-agent, and dual-agent groups, respectively (*p* < 0.001), and the recurrence rate was significantly lower for those administered C&U plus terpene (5.2% vs. 13.2%, *p* = 0.002). In the GB cohort, baseline characteristics were also similar in the groups. GB stone resolution rates of >30% were observed in 5.3%, 14.3%, and 34.8% of patients in the no-medication, single-agent, and dual-agent groups, respectively (*p* = 0.028). **Conclusions:** C&U plus terpene was significantly more effective for preventing CBD stone recurrence and achieving GB stone resolution than no medication or single agents.

## 1. Introduction

The migration of gallbladder (GB) stones from the GB to the biliary tree is the main etiology of common bile duct (CBD) stones [1,2], and symptomatic GB stones are accompanied by coexisting CBD stones in 3–16% of patients [3,4]. CBD stones are usually managed with endoscopic procedures, including endoscopic sphincterotomy (EST) [5], endoscopic papillary balloon dilation (EPBD) [6], and stone extraction. After the endoscopic removal of CBD stones, prophylactic cholecystectomy is routinely offered [7], but stones recur in up to 24% of patients [8,9,10].

The preventative role of oral dissolution therapy based on ursodeoxycholic acid (UDCA) or other choleretic agents in recurrent CBD stones after endoscopic removal has not been established. One randomized control trial (RCT) reported that UDCA is marginally effective at preventing CBD stone recurrence [11], and Somerville et al. concluded that terpene plus UDCA and chenodeoxycholic acid (CDCA) are effective dissolution therapies for CBD stones [12]. Although the combination of UDCA, terpene, and endoscopic biliary stenting was found to be an effective treatment for CBD stones [13], two RCTs showed that adding UDCA to endoprosthetic treatment did not reduce CBD stone size [14,15]. Furthermore, two other studies demonstrated that UDCA and/or terpene did not prevent CBD stone recurrence [16,17].

Nonsurgical management, such as dissolution therapy, is an option for patients with symptomatic GB stones who are either unable or unwilling to undergo cholecystectomy, but dissolution therapy is associated with an overall low curative success rate and a high probability of stone recurrence [18,19,20]. Although the effectiveness of dissolution therapy was not significant, one study found that UDCA had a small (<20 mm) gallstone dissolution efficacy of 30–50% [21]. In addition, a meta-analysis of 23 RCTs reported GB stone dissolution rates of 37% for ursodiol and 62.8% for CDCA plus UDCA (C&U) combination therapy [18]. Other studies have confirmed higher GB stone dissolution rates for combination therapy than monotherapy. In one study, C&U and UDCA achieved complete small stone dissolution rates of 52% and 24%, respectively [22], and another study reported complete stone dissolution rates for UDCA alone and UDCA plus menthol of 38% and 53%, respectively [23].

It is important to determine which drugs are more effective at reducing CBD stone recurrence and dissolving GB stones. Therefore, we evaluated the effects of UDCA, C&U, and terpene (alone and in combination) on CBD stone recurrence and GB stone dissolution.

## 2. Materials and Methods

### 2.1. Patients

**Figure 1:**
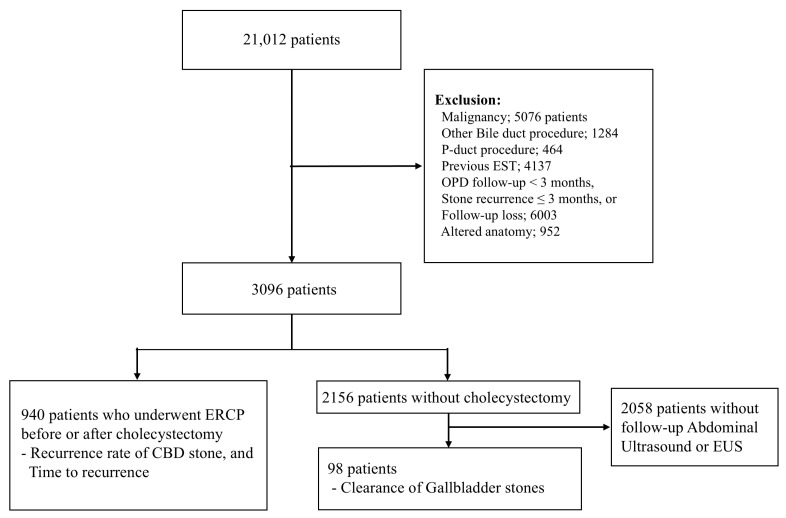
Study flow chart.

The patient flow chart is provided in Figure 1. The records of 21,012 patients who underwent endoscopic retrograde cholangiopancreatography (ERCP) at six referral hospitals during 2011–2015 were screened, retrospectively. Inclusion criteria were ERCP for CBD stone removal and cholecystectomy before or after CBD stone removal by ERCP (the CBD recurrence cohort) or no cholecystectomy and GB stones identified by abdominal ultrasonography or endoscopic ultrasonography (EUS) before and 6 months post-ERCP (the GB cohort). Exclusion criteria were ERCP for malignant obstruction, another bile duct pathology (e.g., benign biliary stricture or intrahepatic duct stone), or a pancreatic duct procedure; EST performed because of previous ERCP; surgically altered anatomy; failure to attend an outpatient follow-up appointment or lost to follow-up within 3 months post-ERCP; or CBD stone recurrence within 3 months post-ERCP.

This study was conducted in accordance with the guidelines of the Declaration of Helsinki (revised in 2013), and the study protocol was approved beforehand by our Institutional Review Board (Approval No. 2021-11-011).

### 2.2. Patient Cohorts

The CBD recurrence cohort included 940 patients who underwent cholecystectomy before or after ERCP. ERCP-related CBD stone recurrence factors, viz. periampullary diverticulum, bile duct stenosis, bile duct diameter, bile duct angle (as defined by Keizman et al. [24,25]), procedure type (EST, EPBD, and mechanical lithotripsy), type of CBD stone removed, and patient data (sex, age, body mass index, and co-morbidities), were evaluated. Recurrent CBD was defined as confirmation by computed tomography, magnetic resonance cholangiopancreatography, or ERCP.

The GB cohort was composed of 98 patients who did not undergo cholecystectomy but underwent ultrasonography before and 6 months after ERCP. The same ultrasound modality (transabdominal or endoscopic) was used pre- and post-ERCP. We assumed that CBD stones and the remaining GB stones were of the same type. Patient data were recorded, and ultrasound images were used to calculate changes in GB stone sizes post-ERCP.

### 2.3. Medication Groups

Recorded patient usages of UDCA (Ursa^®^, Daewoong Pharm. Co., Ltd., Seoul, Republic of Korea), C&U (CnU^®^, Myungmoon Pharm. Co., Ltd., Seoul, Republic of Korea, composed of CDCA 114 mg and UDCA 114 mg), and terpene (Rowahcol^®^, Pharmbio Korea Co., Ltd., Seoul, Republic of Korea) were as follows: UDCA 600 mg/day (200 mg three times daily after meals), C&U 750 mg/day (250 mg three times daily after meals), and terpene 3 capsules daily (1 capsule three times daily before meals). Medication use was defined as being prescribed medication for ≥3 months during follow-up. Patients whose medication regimen changed during follow-up were classified according to the medication received for the longest time. Patients were divided into three groups: no medication, single agents (UDCA, C&U, or terpene), or dual agents (UDCA or C&U plus terpene).

### 2.4. GB Stone Resolution Rate

GB stone resolution was defined based on the percentage change in GB stone size from baseline to 6 months post-ERCP, as determined by ultrasonography. When distinct GB stones were present, stone sizes were measured using the ultrasound length measurement tool, and gallstone size was recorded as the maximum diameter of the largest stone. When only gallbladder sludge was present, sludge volume was calculated using 4/3π × r^3^ (r = radius).

### 2.5. Statistical Analysis

The analysis was performed using IBM SPSS Statistics (version 21.0, IBM Corp., Armonk, NY, USA). Categorical data were expressed as frequencies and percentages, and the significances of intergroup differences were determined using the chi-square test. Continuous data were expressed as means ± standard deviations, and the significances of intergroup differences were determined using the independent Student *t*-test. Statistical significance was accepted for *p* values < 0.05. Univariate and multivariate analyses were conducted to identify factors associated with CBD stone recurrence. Variables with *p* values of <0.100 via univariate analysis were included in the multivariate analysis. Kaplan–Meier plots were used to determine cumulative recurrence rates, and recurrence rates were compared using the log-rank test.

## 3. Results

### 3.1. Baseline Characteristics

Table 1 summarizes the baseline characteristics of the 940 patients between the no-medication group and the medication group in the CBD recurrence cohort. Baseline characteristics were similar between two groups, except for hypertension rates (no medication vs. medication: 49.5% vs. 40.9%, *p* = 0.032), diabetes (27.1% vs. 20.2%, *p* = 0.039), dyslipidemia (14.6% vs. 8.0%, *p* = 0.006), bile duct angle (137.5° ± 18.1° vs. 141.9° ± 19.5°, *p* = 0.005), and mechanical lithotripsy (19.2% vs. 27.3%, *p* = 0.021).

Table 2 summarizes the baseline characteristics of the 747 patients between the single-agent group and the dual-agent group in the CBD recurrence cohort. Baseline characteristics were similar between the two groups, except for EST + EPBD rates (single agent vs. dual agent: 55.6% vs. 30.2%, *p* < 0.001), mechanical lithotripsy (37.2% vs. 20.5%, *p* < 0.001), brown pigment stone (56.2% vs. 63.2%, *p* = 0.026), and cholesterol stone (21.4% vs. 13.0%, *p* = 0.004).

### 3.2. CBD Stone Recurrence

CBD stone recurrence rates differed significantly between the no-medication group and the medication group (no medication vs. medication: 41.5% vs. 11.0%, *p* < 0.001) and within a year (12.4% vs. 5.2%, *p* < 0.001) (Table 3). Time to recurrence was similar in the groups (24.0 ± 18.6 vs. 23.3 ± 22.3 months, *p* = 0.842). Follow-up duration was significantly longer in the no-medication group (37.7 ± 33.7 vs. 26.0 ± 30.7 months, *p* < 0.001).

**Figure 2:**
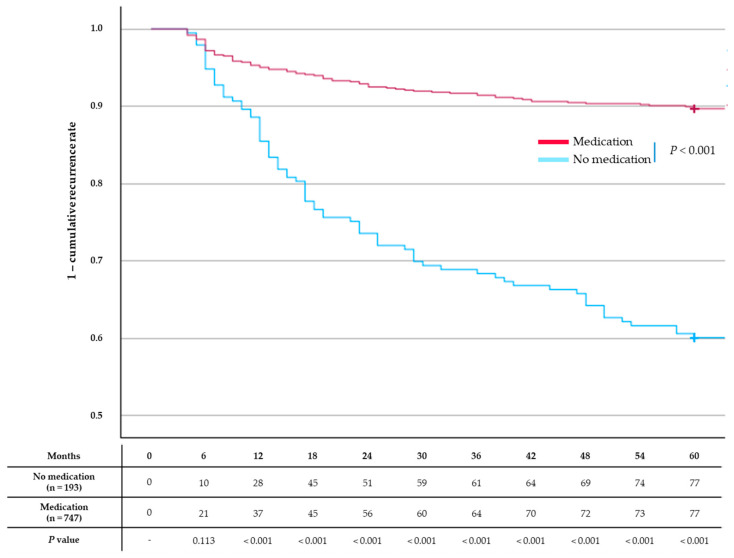
Factors associated with common bile duct stone recurrence. The no-medication group had a significantly higher cumulative recurrence rate than the single-agent or dual-agent groups (*p* < 0.001). However, no significant difference was observed between the two groups at 6 months.

Recurrence rates were significantly lower in the medication group than in the no-medication group from 12 months (14.5% vs. 5.0%, *p* < 0.001) to 60 months (39.9% vs. 10.3%, *p* = 0.002) (Figure 2).

However, CBD stone recurrence rates did not differ significantly between the single-agent group and the dual-agent group (single agent vs. dual agent: 12.7% vs. 9.8%, *p* = 0.198) and within a year (4.6% vs. 5.7%, *p* = 0.509) (Supplementary Table S1). This analysis was performed in response to the diverse range of drugs and their combinations. Time to recurrence was similar in the groups (22.4 ± 17.2 vs. 24.2 ± 26.2 months, *p* = 0.708). Also, follow-up duration was similar in the groups (25.5 ± 29.2 vs. 26.3 ± 31.8 months, *p* = 0.724).

**Figure 3:**
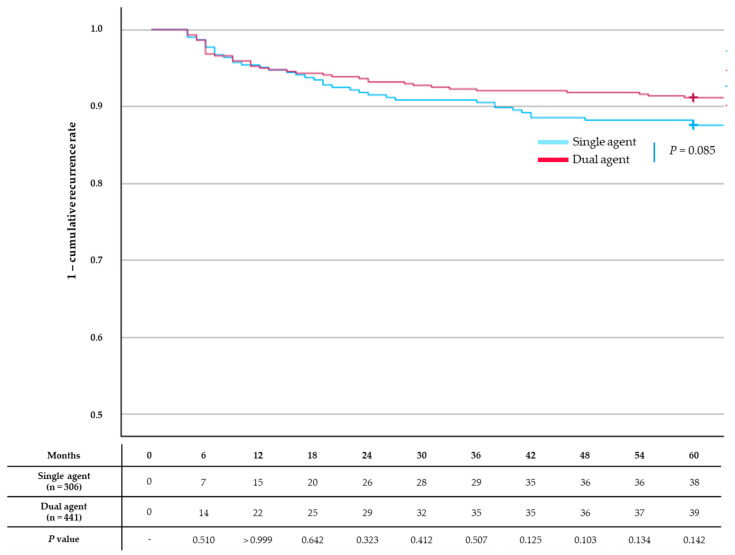
Kaplan–Meier curves for common bile duct stone recurrence. Recurrence rates did not differ significantly between the single-agent group and the dual-agent group (*p* = 0.085).

Recurrence rates did not differ significantly between the single-agent group and the dual-agent group (12.7% vs. 9.8%, *p* = 0.085) (Figure 3).

Subgroup analysis revealed that CBD stone recurrence rates for the medication regimens differed (UDCA vs. C&U vs. terpene vs. UDCA + terpene vs. C&U + terpene: 11.5% vs. 13.3% vs. 19.4% vs. 14.0% vs. 5.2%, *p* = 0.016) (Table 4). A comparative analysis of CBD stone recurrence across various medication subgroups was conducted. In this analysis, the combination of C&U and terpene demonstrated a statistically significant difference compared to other treatments (Supplementary Figure S1). The mean duration of follow-up was similar between the medication regimens (*p* = 0.655). Time to recurrence was significantly different in the medication subgroups (21.2 ± 18.9 vs. 31.8 ± 12.9 vs. 11.2 ± 6.8 vs. 29.2 ± 28.6 vs. 9.6 ± 6.8 months, *p* = 0.011). In the comparative analysis of time to recurrence across various medication subgroups, C&U, as well as the combination of C&U and terpene, demonstrated a statistically significant difference compared to other treatments (Supplementary Figure S2). Also, the duration of medication use was significantly different in the groups (11.1 ± 17.7 vs. 11.7 ± 16.2 vs. 5.1 ± 6.4 vs. 8.5 ± 12.8 vs. 9.0 ± 10.9 months, *p* < 0.001). In the comparison of the duration of medication use across medication subgroups, terpene and UDCA showed a statistically significant difference when compared to other treatments (Supplementary Figure S3).

**Figure 4:**
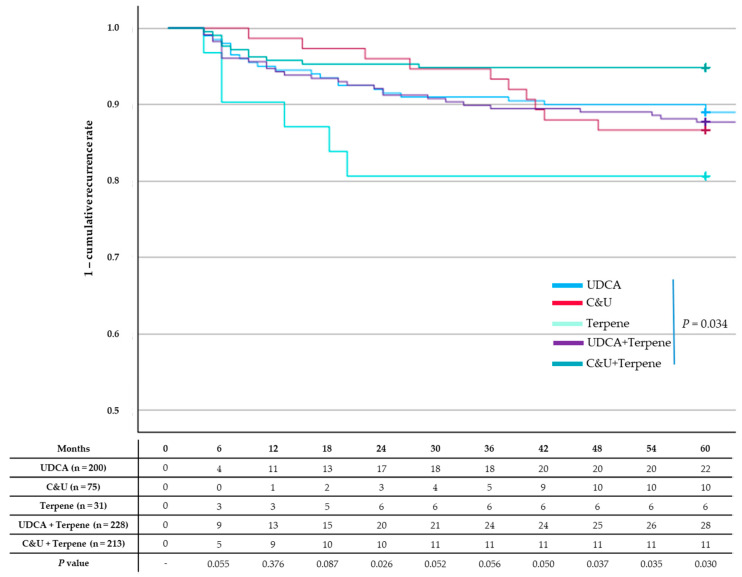
Kaplan–Meier curves for common bile duct stone recurrence. Compared to the other medication groups, the chenodeoxycholic acid and ursodeoxycholic acid (C&U) plus terpene group had significantly lower cumulative recurrence rates (*p* = 0.034).

Recurrence rates were significantly lower in the C&U plus terpene group than in the other medication groups (*p* = 0.034) (Figure 4).

Age, rates of hypertension, periampullary diverticulum, bile duct diameter, bile duct angle, EST, EST + EPBD, EST + EPLBD, mechanical lithotripsy, brown pigment stones, medication, and medication duration in the recurrence and no-recurrence groups were significantly different (Supplementary Table S2). Significant risk factors for recurrence as determined by multivariate regression analysis were age > 70 years (hazard ratio [HR], 2.041; 95% confidence interval [CI], 1.313–3.172; *p* = 0.002), bile duct diameter > 13 mm (HR, 1.802; 95% CI, 1.177–2.759; *p* = 0.007), EST (HR, 0.445; 95% CI, 0.199–0.995; *p* = 0.049), EST + EPBD (HR, 2.061; 95% CI, 1.267–3.352; *p* = 0.004), single agents (HR, 0.122; 95% CI, 0.069–0.215; *p* < 0.001), and dual agents (HR, 0.109; 95% CI, 0.064–0.188; *p* < 0.001) (Table 5).

### 3.3. GB Stone Resolution

**Figure 5:**
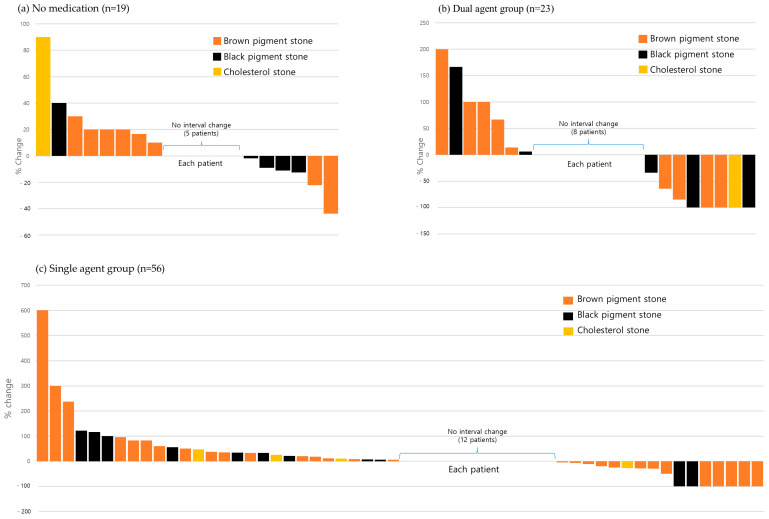
Gallbladder stone resolution by medication group and stone type. Percentage changes in stone size are shown for patients in (**a**) the no-medication group, (**b**) the dual-agent group, and (**c**) the single-agent group. Complete stone resolution was achieved in 7 patients (12.5%) in the single-agent group and 5 patients (21.7%) in the dual-agent group.

Baseline characteristics and GB stone resolution results were not significantly different between medication groups in the GB cohort (Table 6). The resolution rate was highest for brown pigment stones, followed by black pigment stones. Mean medication durations were similar for the single-agent and dual-agent groups (12.0 ± 20.8 vs. 11.1 ± 15.3 months, *p* = 0.870). However, the proportions of patients with a stone resolution rate of >30% were significantly different in the medication groups (no medication vs. single agent vs. dual agent: 5.3% vs. 14.3% vs. 34.8%, *p* = 0.028). Complete resolution was achieved in 12.5% of patients (7/56) in the single-agent group and 21.7% of patients (5/23) in the dual-agent group (Figure 5).

## 4. Discussion

This study shows that stone dissolution medications lowered CBD stone recurrence rates from 41.5% with no medication to 12.7% for single-agent and 9.8% for dual-agent C&U plus terpene therapy treatments. Subgroup analysis revealed that the CBD stone recurrence rate was significantly lower for C&U plus terpene than all other medication regimens combined (5.2% vs. 13.5%). The overall CBD recurrence rate of 17.2% was in line with previously reported recurrence rates of 1–20% [26]. Notably, dual-agent therapy was more effective than single-agent treatment or no medication for GB stone resolution; these treatments achieved > 30% stone reductions in 34.8%, 14.3%, and 5.3% of patients, respectively.

Medication use significantly reduced the risk of CBD stone recurrence, with HRs of 0.122 and 0.109 for single-agent or double-agent treatment, respectively, versus no therapy. In addition, the study showed an age > 70 years (HR, 2.041), bile duct diameter >13 mm (HR, 1.802), EST (HR, 0.445), and EST + EPBD (HR, 2.061) as risk factors of CBD stone recurrence. Previously reported risk factors were age, a large CBD diameter, CBD angle, periampullary diverticulum, EST, mechanical lithotripsy, biliary stenting, and remnant GB [27]. Periampullary diverticulum was not a risk factor via multivariate analysis in the current study, and mechanical lithotripsy was associated with the risk of CBD recurrence via univariate analysis but not multivariate analysis. When we set a cut-off value of 13 mm, according to receiver operating characteristic (ROC) curve analysis (area under the curve [AUC] of 0.610 and highest sensitivity and specificity at a mean bile duct diameter of 12.3 mm), a CBD diameter of >13 mm was also a risk factor for CBD recurrence via univariate but not multivariate analysis. When we set a cut-off value of 145° for the CBD angle based on the findings of another study [28], neither univariate nor multivariate analysis showed an association with CBD stone recurrence, and ROC analysis produced an AUC of 0.557. A large CBD diameter and periampullary diverticulum are associated with cholestatic effects. Furthermore, after ERCP, the possibility of reflux cholangitis increases because of EST, and cholestasis of bile promotes CBD stone recurrence. However, medications such as UDCA or CDCA that enhance bile acid synthesis [29] reduce bile reflux, and thus, stone recurrence.

Combining medications can have additive or synergistic effects on the prevention of CBD stone recurrence. Several studies have demonstrated that UDCA plus CDCA is more effective than either agent alone at dissolving gallstones [21,22,30]. Furthermore, the percentage of cholelitholytic bile acids in bile was nearly 80% for the combination and only ~65% for UDCA alone. Achieving these beneficial changes in bile acid composition is not feasible by administering UDCA or CDCA alone. Specifically, high UDCA doses reduce bile CDCA levels, and thus, reduce cholesterol solubilization into micelles [22]. When CDCA is administered alone, cholesterol does not dissolve in the liquid crystal phase, and bile may remain cholesterol-saturated, even at high CDCA doses, which are poorly tolerated [22]. Terpene alone also has a limited ability to dissolve gallstones, [13] but when combined with CDCA, it provided complete or partial gallstone dissolution in 41% of patients over 6 months [31]. In addition, for patients with CBD stones, three terpene capsules daily resulted in stone dissolution (complete or partial) in 46% of patients after 6 months and 67% of patients after 1 year [32]. Given that combining stone dissolution medications provides effective treatment for established GB or CBD stones, it is likely that combinations are also effective at preventing CBD stone formation.

Several studies have demonstrated gut microbiota dysbiosis after cholecystectomy [33,34] and in patients with GB stones [35], which resulted in studies on the effectiveness of stone dissolution therapy for gut dysbiosis. UDCA treatment restored gut microbiota in patients with non-alcoholic steatohepatitis or primary biliary cholangitis [36,37]. In another study, gut microbiota dysbiosis was partially reversed by C&U treatment in patients with GB stones [35]. Moreover, a recent study that analyzed changes in bile metabolism and composition after UDCA treatment found that UDCA might play a protective role related to gut microbiota in patients with CBD stones [38]. Thus, dissolution therapy may reduce CBD stone recurrence even after therapy by altering the gut microbiome. Moreover, although the effects of terpene on gut dysbiosis have not been evaluated, it might have similar protective effects.

Biliary stones are classified as cholesterol, black pigment, or brown pigment stones. Pigment stones are more common in East Asia and cholesterol stones are most common in Western countries [39,40], and understanding the mechanisms responsible for their formation is essential for the optimal use of stone-dissolving medicines. Cholesterol stones form mainly when bile concentrations of cholesterol are elevated, and all medications used for biliary stones (UDCA, CDCA, and terpene) reduce bile cholesterol levels by controlling HMG-CoA reductase [27,41]. On the other hand, pigment stones originate from increased unconjugated bilirubin levels when the hepatic glucuronic acid concentration is diminished. Reductions in glucuronic acid have many causes, including bacterial β-glucuronidase activity [11]. Regarding treatments, terpene is metabolized to menthol-glucuronic acid, which can combine with unconjugated bilirubin to reduce pigment stone formation. Recurrent CBD stones after EST are usually brown pigment stones [42]. Terpene is the only medication that can suppress bile glucuronic acid reductions and decrease bile cholesterol saturation. Thus, terpene alone might be considered to be capable of resolving or preventing biliary stones. However, clinical data indicate that terpene monotherapy is inadequate [12,23]. Furthermore, brown pigment stones are associated with bacterial ascending cholangitis, which can be caused by EST after ERCP. The use of UDCA or CDCA to enhance bile acid synthesis would be expected to reduce recurrent brown pigment stone formation by decreasing reflux cholangitis, and UDCA-CDCA combination therapy would be expected to be more effective for biliary stone resolution or prevention than a single therapy treatment with either agent. In a study of CBD stones, the complete dissolution rate was 42% for terpene monotherapy and 73% for terpene plus CDCA or UDCA [12], and in a study of GB stones, the complete resolution rate was 37.5% for UDCA monotherapy and 53% for UDCA plus terpene [23]. It has been reported that terpene is more effective when used in combination therapy, and that CDCA and UDCA function more effectively when combined [43]. In the current study, we found that C&U plus terpene was more effective at preventing CBD recurrence than the other treatments. Numerous studies have shown that UDCA, CDCA, and terpene in various combinations act additively or synergistically, and our results suggest that they provide the best CBD stone prevention when combined.

The duration of medication use required to prevent CBD stone recurrence has not been established, though theoretically, treatment should be continued for life. However, this would be difficult and not cost-effective. After initial complete GB stone resolution, stones recur in around 10% of patients during the year following medication discontinuation and in up to 50% of patients by 5 years [44], and the recurrence rate is even higher in patients who do not achieve complete GB stone resolution. Small stone particles or bile sludge may remain after apparent complete CBD stone removal by ERCP, which highlights the importance of determining the appropriate duration of medication administered to dissolve any remaining stone material. Our results suggest that 6–7 months is appropriate, and ROC curve analysis showed that a cut-off value of 6.4 months had the greatest sensitivity and specificity for preventing CBD stone recurrence (AUC, 0.605; sensitivity, 0.353; specificity, 0.773). Also, after removing CBD stone(s) by ERCP, clinical practice guidelines recommend cholecystectomy when GB stone(s) are confirmed upon imaging. However, in patients with significant co-morbidities or other reasons to avoid surgery, medications could be continued as an alternative treatment. Additionally, CBD dilation after cholecystectomy in young patients may be another risk factor for CBD stones [45].

Our study has several limitations. First, due to its retrospective design, medication duration was not controlled, and some differences in group baseline characteristics were detected. Furthermore, the adverse effects of the medications could not be thoroughly investigated. In particular, medication durations differed markedly between patients, and some patients changed medications during the follow-up period, which introduced potential bias. On the other hand, a large cohort of patients was enrolled to reduce the effects of bias. Second, patients probably stopped taking medications during follow-up, and thus, we could not determine how long medication effects persisted after discontinuation. However, beneficial effects probably persisted due to improvements in gut dysbiosis, especially if microlithiasis was completely removed by medications. Third, the number of patients enrolled in the GB cohort was too small to allow for definitive conclusions. Nonetheless, it can be assumed that medication was effective, and the results obtained provide ample evidence to support the design of future large-scale studies.

## 5. Conclusions

The combination of CDCA and UDCA plus terpene was more effective at preventing CBD stone recurrence than other medication regimens. Furthermore, terpene-containing dual-agent regimens were more effective at resolving GB stones than no medication or single-agent treatments. Further research is required to confirm our results and improve our understanding of the mechanisms underlying effective CBD stone prevention.

## Figures and Tables

**Table 1:** Baseline characteristics by medication type.

Baseline Characteristics	No Medication *n* = 193	Medication *n* = 747	*p* Value
Sex, male	106 (55.2)	364 (49.0)	0.125
Age (years)	65.4 ± 14.5	64.2 ± 14.9	0.325
BMI (kg/m^2^)	24.1 ± 3.9	24.0 ± 3.6	0.762
Hypertension	95 (49.5)	305 (40.9)	0.032
Diabetes	52 (27.1)	151 (20.2)	0.039
Dyslipidemia	28 (14.6)	60 (8.0)	0.006
Periampullary diverticulum	73 (37.8)	247 (33.2)	0.232
Bile duct diameter (mm)	12.5 ± 4.9	13.1 ± 6.0	0.117
Bile duct angle (°)	137.5 ± 18.1	141.9 ± 19.5	0.005
Procedure			
EST	188 (97.4)	710 (95.0)	0.157
EST + EPBD	81 (42.0)	303 (40.6)	0.723
EST + EPLBD	30 (15.5)	124 (16.6)	0.724
Mechanical lithotripsy	37 (19.2)	203 (27.3)	0.021
Type of removed CBD stone			
Brown	106 (55.2)	446 (60.4)	0.229
Black	56 (29.2)	172 (23.3)	0.083
Cholesterol	30 (15.6)	121 (16.4)	0.825
Duration of medication use (months)	–	9.5 ± 14.0	-

Data are mean ± standard deviation, number, or number (percentage). BMI, body mass index; CBD, common bile duct; EPBD, endoscopic papillary balloon dilation; EPLBD, endoscopic papillary balloon dilation (≥12 mm); EST, endoscopic sphincterotomy.

**Table 2:** Baseline characteristics by medication type.

Baseline Characteristics	Single Agent *n* = 306	Dual Agent ^#^ *n* = 441	*p* Value
Sex, male	147 (48.0)	217 (49.7)	0.664
Age (years)	64.8 ± 15.2	63.8 ± 14.6	0.359
BMI (kg/m^2^)	24.0 ± 3.4	24.1 ± 3.7	0.835
Hypertension	136 (44.6)	169 (38.3)	0.087
Diabetes	71 (23.2)	80 (18.1)	0.090
Dyslipidemia	18 (5.9)	42 (9.5)	0.074
Periampullary diverticulum	99 (32.6)	148 (33.7)	0.744
Bile duct diameter (mm)	13.2 ± 5.3	13.1 ± 6.4	0.686
Bile duct angle (°)	143.2 ± 17.3	141.0 ± 20.9	0.114
Procedure			
EST	292 (95.4)	418 (94.8)	0.692
EST + EPBD	170 (55.6)	133 (30.2)	<0.001
EST + EPLBD	45 (14.7)	79 (17.9)	0.247
Mechanical lithotripsy	113 (37.2)	90 (20.5)	<0.001
Type of removed CBD stone			
Brown	168 (56.2)	278 (63.2)	0.026
Black	67 (22.4)	105 (23.9)	0.541
Cholesterol	64 (21.4)	57 (13.0)	0.004
Duration of medication use (months)	10.6 ± 16.6	8.7 ± 11.9	0.086

Data are mean ± standard deviation, number, or number (percentage). ^#^ Dual agent = terpene plus UDCA (ursodeoxycholic acid) or terpene plus C&U (chenodeoxycholic acid [CDCA]). BMI, body mass index; CBD, common bile duct; EPBD, endoscopic papillary balloon dilation; EPLBD, endoscopic papillary balloon dilation (≥12 mm); EST, endoscopic sphincterotomy.

**Table 3:** Common bile duct stone recurrence results by medication type.

Recurrence Results	No Medication *n* = 193	Medication *n* = 747	*p* Value
Recurrence	80 (41.5)	82 (11.0)	<0.001
Recurrence within 1 year	24 (12.4)	39 (5.2)	<0.001
Recurrence frequency 1/2/3+	51/21/8	50/22/10	0.891
Time to recurrence (months)	24.0 ± 18.6	23.3 ± 22.3	0.842
Duration of follow-up (months)	37.7 ± 33.7	26.0 ± 30.7	<0.001

Data are mean ± standard deviation, number, or number (percentage).

**Table 4:** CBD stone recurrence results of the medication subgroups.

	UDCA *n* = 200	C&U *n* = 75	Terpene *n* = 31	UDCA + Terpene *n* = 228	C&U + Terpene *n* = 213	*p* Value
Recurrence	23 (11.5)	10 (13.3)	6 (19.4)	32 (14.0)	11 (5.2)	0.016
Duration of follow-up	28.7 ± 32.5	20.3 ± 21.5	17.8 ± 17.9	29.2 ± 33.6	23.3 ± 29.5	0.655
Time to recurrence	21.2 ± 18.9	31.8 ± 12.9	11.2 ± 6.8	29.2 ± 28.6	9.6 ± 6.8	0.011
Duration of medication use	11.1 ± 17.7	11.7 ± 16.2	5.1 ± 6.4	8.5 ± 12.8	9.0 ± 10.9	< 0.001

Data are means ± standard deviations or numbers (percentages). UDCA, ursodeoxycholic acid; C&U, chenodeoxycholic acid plus ursodeoxycholic acid.

**Table 5:** Factors associated with common bile duct stone recurrence.

Factors	HR (95% CI)	*p* Value for Univariate Analysis	HR (95% CI)	*p* Value for Multivariate Analysis
Sex, male	1.064 (0.758–1.495)	0.720		
Age > 70 years	2.457 (1.733–3.484)	<0.001 *	2.041 (1.313–3.172)	0.002 *
BMI ≥ 25 kg/m^2^	0.740 (0.512–1.069)	0.108		
Hypertension	1.498 (1.066–2.106)	0.020 *	0.926 (0.607–1.413)	0.721
Diabetes	1.054 (0.700–1.585)	0.802		
Dyslipidemia	0.991 (0.553–1.776)	0.975		
Periampullary diverticulum	1.452 (1.027–2.055)	0.035 *	1.135 (0.741–1.739)	0.559
Type I	reference	reference		
Type II	0.433 (0.196–0.953)	0.038 *		
Type III	0.585 (0.256–1.338)	0.204		
Type II + III				
Bile duct diameter > 13 mm	1.940 (1.378–2.732)	<0.001 *	1.802 (1.177–2.759)	0.007 *
Bile duct angle < 145°	0.743 (0.529–1.045)	0.088	0.676 (0.447–1.021)	0.063
Procedure				
EST only	0.395 (0.203–0.768)	0.006 *	0.445 (0.199–0.995)	0.049 *
EST + EPBD	3.743 (2.594–5.402)	<0.001 *	2.061 (1.267–3.352)	0.004 *
EST + EPLBD	1.850 (1.238–2.765)	0.003 *		
Mechanical lithotripsy	2.008 (1.402–2.875)	<0.001 *	1.528 (0.923–2.527)	0.099
Type of removed CBD stone				
Brown	reference	reference		
Black	0.704 (0.460–1.077)	0.105		
Cholesterol	0.709 (0.431–1.168)	0.177		
Black + Cholesterol	0.687 (0.482–0.979)	0.038 *	0.871 (0.562–1.352)	0.538
Medication group				
No medication	reference	reference	reference	reference
Single agent	0.206 (0.133–0.321)	<0.001 *	0.122 (0.069–0.215)	<0.001 *
Dual agent	0.153 (0.100–0.234)	<0.001 *	0.109 (0.064–0.188)	<0.001 *
Medication > 7 months	0.555 (0.364–0.847)	0.006 *	1.079 (0.662–1.757)	0.760

Data are means ± standard deviations, numbers, or numbers (percentages). * Statistically significant. Bl, black pigment stone; BMI, body mass index; CBD, common bile duct; CI, confidence interval; EPBD, endoscopic papillary balloon dilation; EPLBD, endoscopic papillary balloon dilation (≥12 mm); EST, endoscopic sphincterotomy; HR, hazard ratio.

**Table 6:** Baseline characteristics and gallbladder stone resolution results by medication group.

	No Medication *n* = 19	Single Agent *n* = 56	Dual Agent *n* = 23	*p* Value
Baseline characteristics				
Sex, male	8 (42.1)	28 (50.0)	12 (52.2)	0.788
Age (years)	66.3 ± 16.5	70.1 ± 14.2	64.8 ± 14.4	0.297
BMI (kg/m^2^)	23.2 ± 6.3	22.9 ± 3.9	24.4 ± 3.3	0.396
Hypertension	9 (47.4)	29 (51.8)	10 (43.5)	0.789
Diabetes	4 (21.1)	13 (23.2)	2 (8.7)	0.326
Dyslipidemia	2 (10.5)	6 (10.7)	1 (4.3)	0.656
Type of removed CBD stone				
Brown/black/cholesterol	11/6/1	37/13/6	11/11/1	0.275
Duration of medication use (months)	-	12.0 ± 20.8	11.1 ± 15.3	0.870
Results				
Stone size change Decrease/no change/increase	6/5/8	16/12/28	8/8/7	0.592
Stone size pre-ERCP (cm)	0.95 ± 0.38	1.10 ± 0.74	1.10 ± 0.77	0.702
Stone size post-ERCP (cm)	1.01 ± 0.50	1.18 ± 0.89	0.85 ± 0.71	0.250
Change in stone size (%)	7.69 ± 27.7	24.1 ± 107.1	–1.3 ± 83.9	0.426
>30% stone resolution	1 (5.3)	8 (14.3)	8 (34.8)	0.028 *†

Data are means ± standard deviations, numbers, or numbers (percentages). * Statistically significant. † *p* = 0.043 for the single-agent vs. dual-agent comparison. BMI, body mass index; CBD, common bile duct.

## Data Availability

The datasets generated and analyzed in the present study are available upon reasonable request to the corresponding author.

## Supplementary Materials

The following supporting information can be downloaded at https://www.mdpi.com/article/10.3390/jcm13237414/s1: Figure S1: Recurrence of the medication subgroups; Figure S2: Time to recurrence of the medication subgroups; Figure S3: Duration of medication use of the medication subgroups; Table S1: Common bile duct stone recurrence results by medication type; Table S2: Baseline characteristics with common bile duct stone recurrence.
