# Supplementary material for: Combinatorial Effects of Terpene, Chenodeoxycholic Acid, and Ursodeoxycholic Acid on Common Bile Duct Stone Recurrence and Gallbladder Stone Dissolution
Source: J Clin Med. 2024 Dec 5;13(23):7414. doi: 10.3390/jcm13237414 (PMC11642399; doi:10.3390/jcm13237414)
Supplement: Supplementary file 1 [file jcm-13-07414-s001.zip › jcm-3274717-supplementary.pdf]

Journal of
Clinical Medicine

MDPI

Supplementary Materials:

Supplementary table S1. Common bile duct stone recurrence results by medication type

Recurrence results	Single-agent n=306	Dual-agent# n=441	*P* value
**Recurrence**	39 (12.7)	43 (9.8)	0.198
**Recurrence within 1 year**	14 (4.6)	25 (5.7)	0.509
**Recurrence frequency 1/2/3+**	22/12/5	28/10/5	0.702
**Time to recurrence (months)**	22.4±17.2	24.2±26.2	0.708
**Duration of follow-up (months)**	25.5±29.2	26.3±31.8	0.724

Data are mean ± standard deviation, number, or number (percentage)
dual-agent = terpene plus UDCA (ursodeoxycholic acid) or terpene plus C&U (chenodeoxycholic acid [CDCA])

Supplementary table S2. Baseline characteristics with common bile duct stone recurrence

Factors	No recurrence n=778	Recurrence n=162	*P* value
**Sex, male**	387 (50.0)	83 (51.6)	0.720
**Age (years)**	63.2±15.1	70.7±11.1	< 0.001*
**Age ≥ 70 years**	303 (39.1)	98 (61.3)	< 0.001*
**BMI (kg/m²)**	24.1±3.7	24.0±3.0	0.682
**BMI ≥ 25 kg/m²**	280 (36.3)	48 (29.6)	0.107
**Hypertension**	318 (40.9)	82 (50.9)	0.019*
**Diabetes**	167 (21.5)	36 (22.4)	0.802
**Dyslipidemia**	73 (9.4)	15 (9.3)	0.975
**Periampullary diverticulum**	253 (32.7)	67 (41.4)	0.034*
Type I	27 (12.4)	13 (22.4)	0.009*
Type II	120 (55.0)	25 (43.1)	0.998
Type III	71 (32.6)	20 (34.5)	0.207
**Bile duct diameter (mm)**	12.6±5.6	14.9±6.4	< 0.001*
**Bile duct diameter > 13 mm**	313 (40.4)	92 (56.8)	< 0.001*
**Bile duct angle**	140.3±20.0	144.4±15.0	0.003*
**Bile duct angle < 145°**	406 (52.5)	73 (45.1)	0.087
**Procedure**			
EST	750 (96.4)	148 (91.4)	0.005*
EST + EPBD	281 (36.1)	103 (63.6)	< 0.001*
EST + EPLBD	112 (14.4)	42 (25.9)	< 0.001*
Mechanical lithotripsy	179 (23.1)	61 (37.7)	< 0.001*
**Type of removed CBD stone**			
Brown	445 (57.9)	107 (66.0)	0.037*
Black	195 (25.4)	33 (20.4)	0.205
Cholesterol	129 (16.8)	22 (13.6)	0.344

**Medication group**			
No medication	113 (14.5)	80 (49.4)	< 0.001*
Single-agent	267 (34.3)	39 (24.1)	0.011*
Dual-agent	398 (51.2)	43 (26.5)	< 0.001*
**Medication duration (months)**	8.8±14.1	5.2±6.3	< 0.001*
**Medication > 7 months**	256 (34.6)	32 (22.7)	0.006*

Data are means ± standard deviations, numbers, or numbers (percentages)
* Statistically significant
Bl, black pigment stone; BMI, body mass index; CBD, common bile duct; EPBD, endoscopic papillary balloon dilation; EPLBD, endoscopic papillary balloon dilation (≥12mm); EST, endoscopic sphincterotomy.

**Medications**	**UDCA** n=200	**C&U** n=75	**Terpene** n=31	**UDCA + Terpene** n=228	**C&U + Terpene** n=213
**UDCA** n=200	***P* value**	0.677	0.243	0.434	0.019*
**C&U** n=75	0.677	***P* value**	0.551	0.879	0.019*
**Terpene** n=31	0.243	0.551	***P* value**	0.421	0.012*
**UDCA + Terpene** n=228	0.434	0.879	0.421	***P* value**	0.002*
**C&U + Terpene** n=213	0.019*	0.019*	0.012*	0.002*	***P* value**

Supplementary figure S1. Recurrence of the medication subgroups
* Statistically significant
UDCA, ursodeoxycholic acid; C&U, chenodeoxycholic acid plus ursodeoxycholic acid.

**Medications**	**UDCA** n=200	**C&U** n=75	**Terpene** n=31	**UDCA + Terpene** n=228	**C&U + Terpene** n=213
**UDCA** n=200	***P* value**	0.739	< 0.001*	0.044*	0.003*
**C&U** n=75	0.739	***P* value**	< 0.001*	0.321	0.030*
**Terpene** n=31	< 0.001*	< 0.001*	***P* value**	< 0.001*	0.211
**UDCA + Terpene** n=228	0.044*	0.321	< 0.001*	***P* value**	0.270
**C&U + Terpene** n=213	0.003*	0.030*	0.211	0.270	***P* value**

Supplementary figure S2. Time to recurrence of the medication subgroups
* Statistically significant
UDCA, ursodeoxycholic acid; C&U, chenodeoxycholic acid plus ursodeoxycholic acid.

**Medications**	**UDCA** n=200	**C&U** n=75	**Terpene** n=31	**UDCA + Terpene** n=228	**C&U + Terpene** n=213
**UDCA** n=200	***P* value**	0.043*	0.235	0.500	0.060*
**C&U** n=75	0.043*	***P* value**	0.007*	0.183	< 0.001*
**Terpene** n=31	0.235	0.007*	***P* value**	0.137	0.721
**UDCA + Terpene** n=228	0.500	0.183	0.137	***P* value**	0.026*
**C&U + Terpene** n=213	0.060*	< 0.001*	0.721	0.026*	***P* value**

Supplementary figure S3. Duration of medication use of the medication subgroups
* Statistically significant
UDCA, ursodeoxycholic acid; C&U, chenodeoxycholic acid plus ursodeoxycholic acid.
